# Investigating the Association between the Autophagy Markers LC3B, *SQSTM1/p62*, and DRAM and Autophagy-Related Genes in Glioma

**DOI:** 10.3390/ijms25010572

**Published:** 2024-01-01

**Authors:** Farheen Danish, Muhammad Asif Qureshi, Talat Mirza, Wajiha Amin, Sufiyan Sufiyan, Sana Naeem, Fatima Arshad, Nouman Mughal

**Affiliations:** 1 Department of Pathology, Dow International Medical College, Dow University of Health Sciences, Karachi 75300, Pakistan; farheen.danish@duhs.edu.pk (F.D.); fatima.arshad@duhs.edu.pk (F.A.); 2 Departments of Research & Molecular Medicine, Ziauddin University, Karachi 75600, Pakistan; deanresearch@zu.edu.pk; 3 Departments of Biological and Biomedical Sciences, Aga Khan University, Karachi 74800, Pakistan; wajiha.amin2@aku.edu (W.A.); sufiyan.ibrahim@aku.edu (S.S.); sana.naeem@aku.edu (S.N.)

**Keywords:** autophagy, autophagy-related genes (ATGs), high-grade gliomas (HGGs), low-grade gliomas (LGGs), *LC3B*, *SQSTM1/p62*, *DRAM*

## Abstract

High-grade gliomas are extremely fatal tumors, marked by severe hypoxia and therapeutic resistance. Autophagy is a cellular degradative process that can be activated by hypoxia, ultimately resulting in tumor advancement and chemo-resistance. Our study aimed to examine the link between autophagy markers’ expression in low-grade gliomas (LGGs) and high-grade gliomas (HGGs). In 39 glioma cases, we assessed the protein expression of autophagy markers LC3B, *SQSTM1/p62*, and DRAM by immunohistochemistry (IHC) and the mRNA expression of the autophagy genes PTEN, PI3K, AKT, mTOR, ULK1, ULK2, UVRAG, Beclin 1, and VPS34 using RT-qPCR. LC3B, *SQSTM1/p62*, and DRAM expression were positive in 64.1%, 51.3%, and 28.2% of glioma cases, respectively. The expression of LC3B and *SQSTM1/p62* was notably higher in HGGs compared to LGGs. VPS34 exhibited a significant differential expression, displaying increased fold change in HGGs compared to LGGs. Additionally, it exhibited robust positive associations with Beclin1 (rs = 0.768), UVRAG (rs = 0.802), and ULK2 (rs = 0.786) in HGGs. This underscores a potential association between autophagy and the progression of gliomas. We provide preliminary data for the functional analysis of autophagy using a cell culture model and to identify potential targets for therapeutic interventions.

## 1. Introduction

Central nervous system (CNS) cancers are rare and heterogeneous tumors with diverse biologies and genetics, which account for about 3% of all cancers in the world and are more common in men [1]. Gliomas constitute the most common type of brain tumors, comprising around 24.7% of all primary tumors in the brain and other CNS tumors, and 74.6% of malignant cases [2]. The most prevalent malignant histology is the glioblastoma, making up 14.2% of all tumors and constituting 50.1% of all malignant CNS tumors [3]. The new 2021 WHO CNS 5 classification has divided diffuse gliomas into adult-type and pediatric-type. The basic molecules for the integrated diagnosis of adult diffuse gliomas are *IDH* (Isocitrate dehydrogenase), *p53*, *ATRX* (alpha-thalassemia/mental retardation, X-linked), and *1p/19q* co-deletion. Adult diffuse gliomas (IDH-mutant astrocytomas, IDH-mutant, 1p/19q co-deleted oligodendroglioma, and IDH-wildtype glioblastoma) are diffusely infiltrating brain tumors [4]. High-grade gliomas, predominantly wild-type glioblastomas, are extremely lethal neoplasms with a poor prognosis. Despite maximum neurosurgical resection and adjuvant therapy; temozolamide (TMZ), median survival barely extends to approximately 12 months [5]. Unfortunately, as with other solid tumors, chemo-resistance is one of the major challenges in this regard. Various mechanisms have been described for chemo-resistance. The evasion of apoptosis is one of the mechanisms of tumor progression and chemo-resistance in gliomas [6], probably through either deficiency in BAX or BAK or a gain of Bcl2 or BclX [7]. Some studies have also revealed that the resistance of cancer cells to chemotherapeutic drugs may be due to the up-regulation of autophagy, thereby avoiding apoptosis [8,9].

Autophagy is a cellular degradation pathway for the breakdown and removal of impaired long-lived proteins, as well as the elimination of organelles and pathogens [10]. Moreover, it has an important homeostatic role in maintaining cell viability in stressed or nutritionally deprived states through recycling the cytoplasmic constituent [11]. Thus, it controls the quality and quantity of proteins and organelles. Dysfunctional autophagy contributes to various diseases—cancer being one of them. Nevertheless, in cancer, the role of autophagy is somewhat complicated and controversial. Autophagy is a double-edged sword as it has dual effects on cancer. On the one hand, it promotes tumor cell survival by breaking down macromolecules into smaller components like amino acids, fatty acids, and metabolic substances. Studies have suggested that autophagy is activated in glioblastoma (GBM) as a reaction to pathophysiological challenges like necrosis and an acidic milieu. Hypoxia, a characteristic feature of high-grade gliomas, is responsible for inducing autophagy in these tumors. As the tumor progresses and reacts to therapeutic interventions, cells must adjust their metabolism to endure in hypoxic and nutrient-deficient surroundings; this adjustment is commonly linked to chemotherapy and radiotherapy resistance. This process facilitates tumor growth and viability, contributing to the maintenance of intracellular metabolism. Under unfavorable hypoxic conditions, molecules such as hypoxia-induced factors (HIF2a and HIF2b), BECN1 (Beclin-1), and BNIP3 (BCL2/adenovirus E1B 19KDa interacting protein 2) serve as survival mechanisms, promoting the progression of GBM and its resistance to anticancer treatmentin vivo [12,13,14,15]. Hypoxia causes BECN1 phosphorylation via the HIF-1a/BECN1 signaling pathway [16,17]. Beclin-1 is a key regulatory protein in autophagosome formation, which binds to the class III PI–3 kinase Vps34, thus facilitating progression of the autophagic process [18,19]. Remarkably, in the presence of hypoxia, the initiation of autophagy through BNIP3/BNIP3L serves as a survival mechanism, fostering the progression of GBM and enhancing its resistance to anticancer treatments in vivo [20]. A recent study showed that in U87 cells exposed to hypoxic conditions, the knockdown of HIF1A significantly reduced BNIP3 expression [21]. This implies that tissues with low oxygen levels are prone to show heightened autophagy, indicating that an increased autophagy flux may serve an adaptive function [15,22]. On the other hand, autophagy can also suppress tumor progression in development, as impaired autophagy causes oxidative stress, triggers DNA damage responses, and results in genomic instability, a known cause of tumor initiation. Such dual effects on tumor behavior are most likely context-dependent [23,24,25,26]. Moreover, research has indicated that prolonged hypoxic stress in vitro up-regulates a pro-apoptotic Bcl-2 protein (BNIP3), leading to hypoxia-dependent autophagic cell death (ACD) in GBM cells [27]. In terms of this mechanism, the elevated expression of BNIP3 leads to the release of BECN1 from complexes with Bcl-2 or Bcl-xL, thereby enabling BECN1 to activate autophagy [28]. Hence, autophagy has been found to show conflicting functions in tumor onset and advancement. Thus, the manipulation of autophagy pathways as a means of cell death in cancer has led to the exploration of both inhibitors and inducers. Keeping in mind the contradictory results of autophagy in cancer, we investigated the expression of autophagy markers and autophagy-related genes (ATGs) in our cohort of low- and high-grade gliomas.

## 2. Results

### 2.1. Clinicopathologic Characteristics of Adult Diffuse Glioma Patients

Demographic data of the studied cases showed that the average age of the patients was 43.14 ± 13.25. Of the 39 adult diffuse glioma patients enrolled in this study, 23 (59%) were males and 16 (41%) were females. Histological examination showed that *IDH*-mutated astrocytic tumors were identified as the most frequently diagnosed subtype of adult diffuse gliomas (48.7%), followed by oligodendroglial tumors (30.8%) and glioblastoma *IDH*-wild type (20.5%). When cases were stratified based on histological grades, 15 (38.5%) cases were of grade 4, 13 (33.3%) were of grade 3, and 11 (28.2%) were of grade 2. Moreover, of the 39 cases, 25 (64.1%) showed *IDHR132* mutation, 16 (41%) showed *p53* mutation, and 21 (53.8%) showed *ATRX* loss (mutation), as shown in Table 1.

### 2.2. Evaluation of Autophagy Status Using Immunohistochemical Analysis of Molecular Markers (LC3B, SQSTM1/p62, and DRAM)

**Figure 1:**
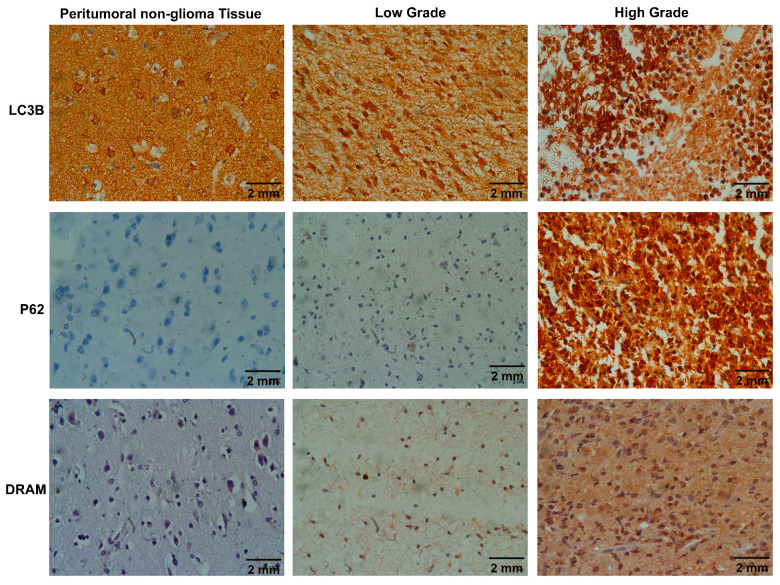
Representative images of autophagy markers *LC3B*, *SQSTM1/p62*, and *DRAM* immunohistochemical stains in peritumoral non-glioma tissue, low-grade glioma (LGG) and high-grade gliomas (HGG) at ×400. Peritumoral non-glioma tissues and LGGs showing negative staining for autophagy markers while HGGs displaying strong dotted or punctuate cytoplasmic staining for *LC3B* and *SQSTM1/p62* and diffuse cytoplasmic staining for *DRAM*.

Autophagy-related protein expression of *LC3B*, *SQSTM1/p62*, and DRAM was performed in 39 LGG and HGG cases to assess the autophagy status in the current study. Diffuse cytoplasmic and nuclear expression of *LC3B* was found in all cases. Nonetheless, punctate strong cytoplasmic expression of *LC3B* and *SQSTM1/p62* was categorized as a positive result, and the presence of positive cytoplasmic staining for *DRAM* was also considered a positive outcome. Our result showed a strong punctate cytoplasmic expression of *LC3B*, *SQSTM1/p62*, and HGGs compared to LGGs and peritumoral non-glioma tissue. *DRAM* also exhibited positive and negative cytoplasmic staining (Figure 1).

### 2.3. Correlation of Clinicopathological Features and Autophagy Markers

Among the 39 glioma cases analyzed, 23 (59%) cases of HGGs showed a positive expression of LC3B, whereas *SQSTM1/p62* demonstrated positive expression in all 20 (100%) HGG cases. DRAM overexpression was detected in the cytoplasm of 11 (28.2%) cases, encompassing both LGGs and HGGs (Table 2).

Consequently, elevated levels of *LC3B* and *SQSTM1/p62* were frequently observed in HGGs, with *p*-values of 0.001 and <0.001, respectively. However, there was no significant correlation between *DRAM* overexpression and histologic grades (Table 3). Furthermore, in this study population, autophagy status was considered positive in 19 (48.7%) cases. A statistically significant correlation was detected when comparing the age groups with the autophagy markers. Significant correlations were found between age groups and the autophagy markers *LC3B* (*p* = 0.022) and *SQSTM1/p62* (*p* = 0.017). Moreover, when examining the correlation between autophagy markers and clinicopathologic factors such as gender, histological type, and molecular markers of glioma including *IDH1*, *ATRX*, and *p53*, no statistically significant differences were observed (Table 4).

### 2.4. Analysis of Autophagy-Related Gene (ATG) Expression in High-Grade and Low-Grade Gliomas

**Figure 2:**
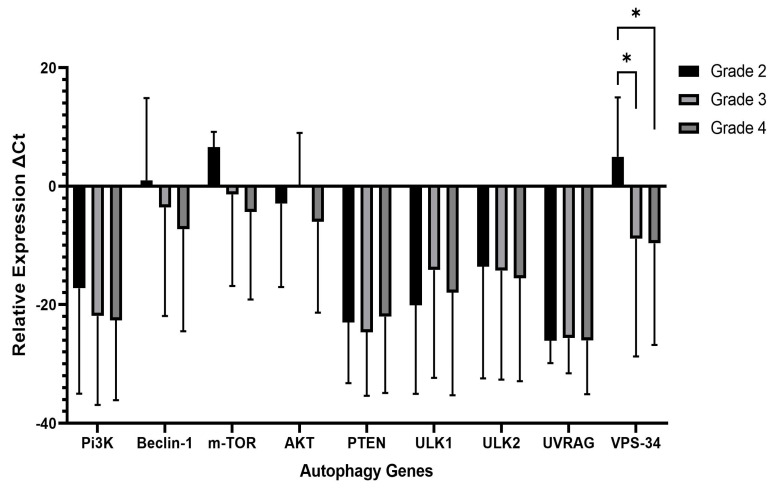
Expression of autophagy-related genes (ATGs) in 2, 3, and 4 WHO grades of diffuse gliomas in 39 patients using housekeeping gene Beta-actin. The Y-axis shows the relative expression of each ATG tested. The line with the asterisk sign shows a significant difference (* *p* < 0.01) in the expression of VPS34 between the various grades of gliomas.

Following the immunohistochemical analysis of well-established autophagy markers, we selected nine autophagy-related genes, including the master regulators and core autophagy genes such as *PTEN*, *PI3K*, *AKT*, *mTOR*, *ULK1*, *ULK2*, *UVRAG*, *Beclin 1*, and *VPS34.* The difference in relative mRNA expression of autophagy-related genes was compared in different grades of 39 glioma cases. We found a statistically significant difference in *VPS34* mRNA relative expressions among grades 2 and 3 (*p* = 0.01) and grades 2 and 4 (*p* = 0.01). Nevertheless, there was no notable difference in the expression of other genes associated with the essential autophagy machinery between LGGs and HGGs. Comparisons of *PI3k* (*p* = 0.495), *AKT* (*p* = 0.85), *PTEN* (*p* = 0.78), *Beclin1* (*p* = 0.217), *ULK1* (*p* = 0.785), *ULK2* (*p* = 0.524), and *UVRAG* (*p* = 0.387) between grade 2 and grade 4 showed statistically insignificant differences, as shown in Figure 2. Similarly, the negative autophagy regulator *mTOR* was not significantly differentially expressed between the two glioma grades (*p* = 0.07), but higher expression was noticed in higher grade.

**Figure 3:**
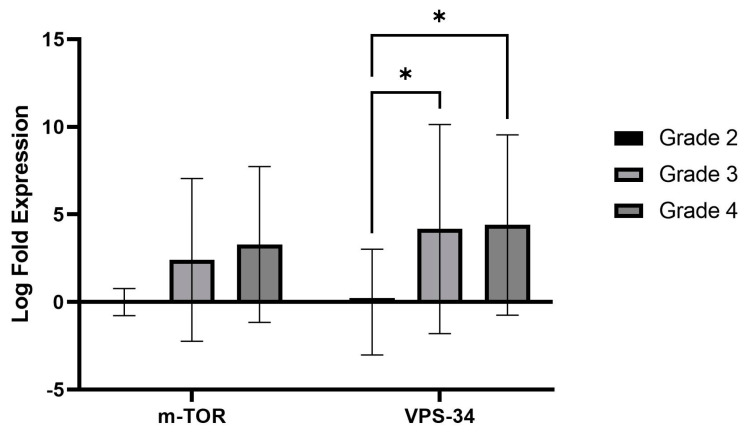
The graph depicts the fold change in the expression of VPS34 and mTOR in the WHO grade 2, 3, and 4 gliomas. The Y-axis represents the logarithmic fold expression. The lines marked with asterisks indicate a notable difference (* *p* < 0.01) in VPS34 expression between low- and high-grade cases.

Next, log2 fold change (FC) was executed using grade 2 gliomas as the reference group. Two genes were selected for log2 fold change (FC) ≥ 2. We found the expression of *VPS34* to be significantly increased in HGGs (*p* = 0.01). Expression of mTOR, a negative regulator of autophagy, is increased in grade 3 and grade 4. However, it did not reach statistical significance (*p* = 0.07), as shown in Figure 3.

Furthermore, we applied the Spearman correlation test to examine the correlation between ATGs in the WHO grade 2, 3, and 4 gliomas. In WHO grade 2, a significant strong positive correlation was observed between *PI3K* and *PTEN*, *ULK2*; *mTOR* and *PTEN*, *ULK1*; *PTEN* and *PI3K*, *m-TOR*, *ULK1*, *ULK 2*, *UVRAG*; *ULK1* and *mTOR*; *ULK 2* and *PI3K*, *PTEN*, *ULK 1*; and *UVRAG* and *PTEN.* A significant, moderate, positive correlation was observed between *BECLIN 1* and *mTOR*, *PTEN* and *BECLIN 1*, *ULK2* and *UVRAG*, *VPS34*; *UVRAG* and *ULK2*; and *VPS34* and *ULK 2* (Table 5).

In grade 3 gliomas, a significant, strong, positive correlation was observed between *PI3K* and *mTOR*, *ULK2*, *UVRAG; mTOR* and *PTEN; PTEN* and *m-TOR*, *ULK 2*, *UVRAG; mTOR* and *PI3K*, *PTEN; ULK1* and *ULK2*, *UVRAG; ULK 2 and PI3K*, *PTEN*, *ULK 1*, *UVRAG*, *VPS34; UVRAG* and *PI3K*, *PTEN*, *ULK1*, *ULK2*, *VPS34*. A significant, moderate, positive correlation was observed between PI3K and *ULK1*; *BECLIN 1* and *mTOR*; *mTOR* and *VPS34*; *PTEN* and *ULK1*; and *ULK1* and *PI3K*, *VPS34* (Table 6).

In WHO grade 4 diffuse gliomas, a significant, strong, positive correlation was observed between *PI3K* and *BECLIN 1*, *ULK2*; *BECLIN 1* and *ULK1*, *ULK2*, *VPS34*; *ULK2* and *ULK1*; *UVRAG* and *ULK2*; and *VPS34* and *ULK2*. A significant, moderate, positive correlation was observed between *PI3K* and *PTEN*, *VPS34*; and *ULK 1* and *VPS34* (Table 7).

## 3. Discussion

Autophagy is primarily a stress response process, and most of body tissues need autophagy to eliminate accumulated damaged organelles and unfolded proteins to maintain normal homeostatic milieu. In cancer, autophagy plays both tumor-suppressing and tumor-promoting roles depending on the specific context; thus, it could influence the prognosis either favorably or adversely. We investigated whether autophagy plays anti-tumoral or pro-tumoral functions in various grades of gliomas.

In this current research, we assessed the immunohistochemical expression of the autophagy markers and the mRNA levels of ATGs in different grades of adult-type diffuse gliomas while also assessing their correlation with clinicopathological parameters. Our investigation revealed a significant connection between the immunohistochemical expression of *LC3B*, *SQSTM1/p62*, and *DRAM*, and the overall autophagy status, particularly tumor grades. Notably, among the 25 positive cases of *LC3B*, only two were observed in the LGGs. Similarly, out of the 20 strongly positive cases of *SQSTM1/p62*, none were found in the LGG category, and this difference achieved statistical significance (as indicated in Table 3). These results are in accordance with other studies, which reported that *LC3B* and *SQSTM1/p62* were highly expressed in high-grade gliomas [29,30,31]. Remarkably, there is a statistically significant association between the autophagy markers *LC3B* and *SQSTM1/p62* (*p* = 0.000), highlighting the interaction between them. These results may be attributed to the active participation of *LC3B* and *SQSTM1/p62* in the process of autophagy, given their roles as essential structural components of the autophagosome [32], and recent literature also revealed that *SQSTM1/p62* overexpression is capable of promoting mitochondrial and classical macroautophagy [33], which promote tumor progression and chemo-resistance. Furthermore, increased expression of *LC3B* and *SQSTM1/p62* in HGGs compared with LGGs may be in response to conferring stress tolerance, which is greater in HGGs, and serves to maintain tumor cell survival [34]. Hypoxia, which is a major hallmark of glioblastoma, is known to induce autophagy in these tumors, which eventually produces a survival mechanism facilitating the breakdown of various cellular components to generate ATP and metabolic precursors to cope with stress, highlighting the crucial role of autophagy in protecting cells against stressful conditions [35]. According to a study conducted by Deng et al., both the mRNA and protein levels of SQSTM1/p62 were found to be elevated in human glioma tissues. Furthermore, it was discovered that the suppression of SQSTM1/p62 had an anti-tumor effect on glioma cells [34]. These findings suggest that high levels of LC3B and SQSTM1/p62 expression, or prompted autophagy, are correlated with advanced tumor grade and aggressiveness. Additionally, these findings support the potential role of autophagy as a tumor enhancer, which further supports the results of the study.

We also looked at another important autophagy modulator, *DRAM1*, in our study and found its overexpression in 28.2% of the cases; it was not significantly associated with tumor grades, but higher expression of *DRAM1* was present in high-grade gliomas when compared with LGGs. *DRAM1* primarily localizes to lysosomes and is frequently downregulated in various human cancers [36]. *DRAM1* enhances lysosomal acidification and facilitates the fusion of lysosomes with autophagosomes, thereby promoting autophagy. Moreover, *DRAM1* plays a pivotal role in governing the association of *SQSTM1/p62* with autophagosomes and its subsequent degradation through autophagy. Therefore, reduced *DRAM1* expression might be associated with decreased *SQSTM1/p62* localization within autophagosome, highlighting the role of *DRAM1* in *SQSTM1/p62*-mediated autophagy. Our observations are in line with the research conducted by Geng et al., which also indicated decreased DRAM1 expression in non-small-cell lung carcinoma, linked with an unfavorable prognosis [37]. However, another study showed high expression of both *DRAM1* and *SQSTM1/p62* in glioblastoma, where they regulate cell migration and invasion and are associated with shorter or poor overall survival [38]. These conflicting results could arise from variations in demographics and sample sizes. Thus, utilizing the expression levels of *LC3B*, *SQSTM1/p62*, and *DRAM1* to assess autophagy status in glioma patients could be potential predictive markers.

While investigating the autophagy gene expression patterns in both LGGs (grade 2) and HGGs (grades 3 and 4), we observed a remarkable and statistically significant up-regulation in the expression of vacuolar protein sorting 34 (*Vps34*), a critical kinase in autophagy. This indicates that the VPS34 gene plays a role in promoting autophagy in gliomas. Previous research has shown that *VPS34* initiates autophagy by interacting with Vps15/Atg14/UVRAG/Beclin1 [39]. Therefore, inhibiting VPS34 has been considered as a potential target for inhibiting autophagy [40,41].

We also evaluated the autophagy upstream pathway, *PI3K/AKT/mTOR.* We recorded higher expression of *mTOR* transcripts in HGGs than LGGs, a negative autophagy regulator, but it did not reach a significant value (*p* = 0.07). The *PI3K/Akt/mTOR* signaling pathway is a frequently disrupted pathway across different cancer types [42,43], and abnormal activation of this pathway has been associated with tumor development, progression, invasion, and metastasis [44] and is indeed activated in glioma cells [45]. Nonetheless, the *PTEN*, *PI3K*, *AKT*, *mTOR*, *Beclin-1*, *UVRAG*, *ULK1*, and *ULK2* genes remained unchanged when compared in both grades. The significant increase in transcriptional expression of VPS34 implies an increased initiation of autophagy, as VPS34 plays a crucial role in the nucleation of autophagosomes. Conversely, the absence of substantial transcriptional alterations in other genes suggests that these components may not be profoundly influenced at the transcriptional level in high-grade gliomas. Nevertheless, it is important to consider the potential involvement of post-transcriptional, post-translational, or alternative regulatory mechanisms. The modulation of autophagy in high-grade gliomas depends on the unique context of these tumors, which are heterogeneous and have diverse genetic and epigenetic profiles. Therefore, autophagy signaling pathways may vary among individual tumors.

Spearman’s correlation between autophagy genes was examined in grades 2, 3, and 4 of glioma cases, and we found a significant positive correlation among different genes. It is essential to highlight that these observed correlations were significant, but whether they have a biological basis remains uncertain. For instance, in grade 2 gliomas, *PTEN* showed a strong positive correlation with autophagy genes, which aligns with a study by Errafiy Rajaa [46]. However, the current study’s absence of *PTEN* correlation in grade 4 highlights the loss of *PTEN*, a hallmark of GBM, and could be due to mutation or promoter methylation of *the PTEN* gene [47,48].

Similarly, in HGGs, the significant positive correlation between *VPS34* and *ULK1*, *ULK2*, *UVRAG*, *Beclin1*, and *PI3K* genes can also be justified biologically, as the literature reports that the *VPS34* kinase forms a stable complex with Beclin1 and p150, serving as a binding partner for *ATG14L*, *UVRAG*, and *AMBRA* [49], which are responsible for the promotion of autophagy. Thus, a significant positive correlation indicates that the expression of autophagy genes tends to change consistently, providing insights into the regulatory connections within autophagic pathways. This understanding may have implications for comprehending the underlying mechanisms influencing tumor progression.

Nevertheless, a limitation of our study is the absence of autophagy flux assessment in formalin-fixed, paraffin-embedded tissue block (FFPE) samples. Elevated expression levels of *LC3B* and *SQSTM1/p62* do not consistently correlate with an overall augmentation in autophagy. They may be attributed to a potential hindrance in autophagy at the later stages of autophagosome processing. Thus, there is a need for a more comprehensive assessment of autophagy, particularly considering the dynamic nature of the process. Moreover, ATG5, ATG12, ATG 7, and ATG 4 are crucial for two conjugation systems (Atg8–Atg4 and Atg12–Atg5) involved in autophagy and would provide valuable information, as these processes contribute to the formation and elongation of autophagosomes, facilitating the degradation and recycling of cellular components. Unfortunately, due to financial constraints, we could not perform this analysis. However, future comprehensive studies utilizing diverse techniques will be invaluable in addressing these limitations.

## 4. Materials and Methods

### 4.1. Patients Selection

Patients with adult diffuse gliomas were enrolled from the Histopathology Department of the Dow Diagnostic Reference and Research Lab (DDRRL) at Dow University of Health Sciences (DUHS). The Institutional Review Board at Dow University of Health Sciences approved the research protocol (Ref: IRB-1150/DUHS/Approval/2018). Clinical information was recorded. The study included patients diagnosed with adult diffuse gliomas of all grades. A total of 50 adult diffuse gliomas were initially included in the study, excluding patients diagnosed with pediatric-type diffuse gliomas and astrocytomas with circumscribed morphology. However, due to the use of multiple immunohistochemical stains and the extraction of nucleic acids from tissue samples, some cases ran out of available tissue. As a result, only 39 patients were finally included in the study. The study aimed to determine the mRNA and protein expression of autophagy-related genes (ATGs) using Quantitative real-time PCR (qPCR) and IHC.

### 4.2. Tissue Processing for Histopathological Analysis

The histopathology department received biopsy specimens of brain tumors from various hospitals and centers in the city through its collection point service. For histopathological examination, every specimen was placed in an automated tissue processor (Thermo Scientific, model EXCELSIOR AS) for 12 hours, where they were exposed to a graded series of alcohol at 70%, 95%, and 100%; followed by xylene; and then paraffin. The paraffin blocks obtained were sliced into sections measuring 4–5 μm thickness for subsequent hematoxylin and eosin (H&E) staining. The slides were deparaffinized before staining, and the sections were treated with xylene, followed by hydration through decreasing concentration of ethanol of 100%, 70%, and 50%. Slides were then cleaned with water before being submerged in H&E for staining. To eliminate excess water from the slide after staining, the sections were submerged in 60%, 80%, and 100% ethanol. Slides were covered by a coverslip with Enthelan^®^ after being submerged in xylene (Merck, Darmstadt, Germany). The Nikon Eclipse E200 optical microscope (Nikon Instruments Inc. in Tokyo, Japan) was used to perform the histopathological examination of the H&E stained slides, and the tumors characterized as adult diffuse gliomas were enrolled in the study. The cases of diffuse gliomas were categorized according to CNS5 classification into distinct subtypes and malignancy grades using histopathological features such as cellularity, atypia, necrosis, micro-vascular proliferation, and mitosis.

### 4.3. Tissue Processing for Immunohistochemical Examination

IHC was carried out using the Autostainer Link 48 (Dako North America Inc., USA, S no AS3006D1307, Carpinteria, CA, USA) on formalin-fixed, paraffin-embedded (FFPE) tissue blocks for *LC3B*, *p62*, and *DRAM* antibodies. Sections were sliced into 3–4 μm, affixed on charged slides (EnVision FLEX visualization systems), and dried for 60 min at 60–70 °C. The slides were then deparaffinized with xylene, rinsed in decreasing ethanol concentrations, and finally rehydrated in distilled H2O. In a preheated water bath, retrieval solution (EnVision Flex Target Retrieval solution, pH 9.0, TRIS HCL) was used for 20 min to facilitate antigen unmasking. To quench endogenous peroxidase activity, slides were immersed in peroxidase blocking solution (EnVision Flex Peroxidase blocking reagent, RTU) for 10 min. TBST (Tris Buffer saline with Tween 20, EnVision Flex wash Buffer) was used for washing; then, sections were incubated for 30–60 min at room temperature with primary antibodies LC3B (ABCAM, ab51520: 1: 800), p62 (Invitrogen, Waltham, MA, USA, clone: SOSTM1: 1: 25), and DRAM (Invitrogen, 1; 25). Subsequently, the sections were subjected to a 30 min treatment at room temperature with a secondary antibody (EnVision Flex/HRP, RTU) after washing with PBS buffer. DAB solution 3, 3-diaminobenzidine tetrahydrochloride solution (EnVision Flex DAB+ chromogen) was applied for 10 min to the sections on the slides to reveal the color of antibody staining and counterstained with hematoxylin. Slides were washed for 10 min in running water. Sections were then dehydrated (in graded alcohol of 80%, 90%, and 100%), cleaned, and cover-slipped using a DPX mounting solution.

### 4.4. Immunohistochemical Evaluation of Autophagy Markers

*LC3B* and *SQSTM1/p62* punctate/dotted cytoplasmic staining were considered as positive [50,51]. The immunopositivity was scored based on the intensity and percentage of positive glioma cells. Immunopositivity of >50% of tumor cells was considered as positive for *LC3B*, whereas for *SQSTM1/p62*, it was considered positive when it exceeded >30% [52]. The intensity of DRAM1 staining was scored on a scale of 0 to 3, where 0 means no staining, 1 means weak staining, 2 means moderate staining, and 3 means strong staining. Percentage scores were assigned on a scale of 1 to 4, where 1 means 0–25% staining, 2 means 26–50% staining, 3 means 51–75% staining, and 4 means 71–100% staining. Each sample was given a score, which was then multiplied to calculate a total value that ranged from 0 to 12. A score of 4 or more was defined as DRAM1 overexpression, while a score of less than 4 was classified as weak or negative expression [53]. The immunohistochemical staining was independently scored by two pathologists at 40× objective magnification; then, discrepancies were discussed on a multi-head microscope and final scores were determined. Autophagy status was considered positive when two out of the three autophagy-associated proteins were detected in each sample [54].

### 4.5. RNA Extraction and cDNA Synthesis

For the RT-qPCR analysis, total RNA was isolated from FFPE blocks via Pure Link FFPE, total isolation (Invitrogen; Thermo Fisher Scientific, Inc., Waltham, MA, USA) according to the manufacturer’s protocols [55]. Subsequently, DNase treatment was carried out for any DNA contamination. This involved combining 1 μg of RNA template with 1 μL of reaction buffer containing MgCl_2_; 1 μL of DNase-I, RNase-free (Thermo Fisher Scientific, Cat. No. EN0521); and nuclease-free water in a 0.2 mL tube. The mixture was incubated in a Master cycler X50a (Eppendorf, Hamburg, Germany) for 30 min at 37 °C. To prevent RNA degradation, following the DNase-I treatment, we introduced 1 μL of 50 mM EDTA, and the samples were incubated at 65 °C for 10 min. The RNA integrity was evaluated with a nanodrop and, subsequently, cDNA was generated according to the manufacturer’s instructions using the Revert-Aid First Strand cDNA Synthesis Kit (Thermo Fisher Scientific, Cat. No. K1612). The resulting cDNA was stored at −20 °C for future applications.

### 4.6. Gene Expression Analysis via Quantitative Real-Time PCR (qPCR)

qPCR was conducted to assess the gene expression levels of *ULK1*, *ULK2*, *Beclin 1*, *UVRAG*, *VPS34*, *PTEN*, *PI3K*, *AKT*, and *mTOR* using a PCR kit (PowerUp™ SYBR™ Green Master Mix (Thermo Fisher Scientific, Cat. No A25742) and primers (Eurofins, Barberton, OH, USA). β-actin, a housekeeping gene, was used for result normalization in the qPCR assay, utilizing the corresponding primer sets. To perform qPCR analysis, cDNA samples were used, and a 10 μL reaction mixture was prepared. The mixture comprised 2 microliter cDNA, one μL primers of both forward and reverse primers, 5 μL of PowerUp™ SYBR™ Green Master Mix, and 1–2 microliter of nuclease-free water. The thermal cycling conditions for the reaction were as follows: an initial 2 min hold at 50 °C, another 2 min hold at 95 °C, and then 40 cycles of denaturation for 15 sec at 95 °C followed by annealing at 60 °C for 1 min. 2^−ΔΔCt^ (Livak’s method) was used to analyze the relative changes in gene expression, and a multivariate ANOVA test was performed to determine statistical significance. Following the differential expression of ATGs, the log2 fold change was calculated. Grade 2 gliomas, considered low grade, were chosen as the baseline group for computing the log2 fold change (FC). Table 8 shows the list of primers that were utilized.

### 4.7. Statistical Analysis

Descriptive statistics was used to express the means with standard deviation. Pearson’s Chi-square test was executed for the association of demographics, clinical pathologic parameters, and molecular markers with tumor type and grade. Multivariate ANOVA test was performed to identify any notable difference in the relative gene expression among all the examined ATGs in 2, 3, and 4 WHO grades of diffuse gliomas, and *p* < 0.05 was considered significant. Spearman’s correlation test was executed to determine the correlation between autophagy genes in grade 2, 3, and 4 gliomas. We performed all the analyses using IBM SPSS version 24 and used a significance threshold of *p* < 0.05 to determine statistical associations.

## 5. Conclusions

In summary, the significantly high expression of autophagic proteins *LC3B* and *SQSTM1/p62*, coupled with increased mRNA expression levels of *VPS34* in high-grade glioma, underscores the connection between autophagy and the advancement of gliomas. Moreover, assessing autophagy status through *LC3B* and *SQSTM1/p62* expression could be a promising prognostic tool for glioma patients. We provide preliminary data for the functional analysis of autophagy using a cell culture model to identify potential targets for therapeutic interventions.

## Figures and Tables

**Table 1:** Clinicopathologic characteristics of adult diffuse glioma patients.

Variables		Values
**Gender**	Male	23 (59%)
	Female	16 (41%)
**Age in years**	Median (Range)	43 (47)
**Histopathological Type**	Astrocytoma (Grade 2–4)	19 (48.7%)
	Oligodendroglioma (2 and 3)	12 (30.8%)
	Glioblastoma (Grade 4)	08 (20.5%)
**Histopathological grade**	2	11 (28.2%)
	3	13 (33.3%)
	4	15 (38.5%)
**Glioma Grade Group**	Low Grade	11 (28.20%)
	High Grade	28 (71.8%)
***IDH 1*, N (%)**	Mutant	25 (64.1%)
**TP53, N (%)**	**Mutant**	16 (41%)
**ATRX, N (%)**	**Mutant**	21 (53.8%)

**Table 2:** Frequency of *LC3B*, *SQSTM1/p62*, *and DRAM* in 2, 3, and 4 WHO grades of adult diffuse gliomas.

		LC3B		P62		DRAM	
**Cases**	**Total**	**Punctate**	**Diffuse**	**High**	**Low**	**Positive**	**Negative**
	39	25 (64.1%)	14 (35.9%)	20 (51.3%)	19 (48.7%)	11 (28.2%)	28 (71.8%)
**Grades**	**G-2** (11)	02 (8%)	09 (64.2%)	00 (0%)	11 (58%)	04 (36.3%)	07 (25%)
	**G-3** (13)	12 (48%)	01 (7.1%)	10 (50%)	03 (15.7%)	03 (27.2%)	10 (35.7%)
	**G-4** (15)	11 (44%)	04 (28.5%)	10 (50%)	05 (26.3%)	04 (36.3%)	11 (39.2%)

**Table 3:** Association of autophagy markers with histological grades.

Grade	LC3B		*p*-Value	P62		*p*-Value	DRAM		*p*-Value
	Punctate	Diffuse		High	Low		Positive	Negative	
**2**	2 (18%)	09 (82%)	0.001 *	0 (0%)	11 (100%)	0.001 *	4 (36%)	07 (64%)	0.760
**3**	12 (92%)	1 (7%)		10 (77%)	3 (23)		3 (23%)	10 (77%)	
4	11 (73%)	4 (27%)		10 (67%)	5 (33%)		4 (27%)	11 (73%)	

* indicates Significant *p*-value.

**Table 4:** Correlation of autophagy markers with clinicopathologic parameters.

Clinicopathologic Parameters		*LC3B*		*p*-Value	*P62*		*p*-Value	*DRAM*		*p*-Value
		Punctate	Diffuse		High	Low		Present	Absent	
**Age**	>45	10 (40%)	11 (79%)	**0.022**	07 (35%)	14 (74%)	**0.017**	05 (45%)	16 (57%)	0.380
	<45	15 (60%)	03 (21%)		13 (65%)	05 (26%)		06 (55%)	12 (43%)	
**Gender**				0.117			0.133			0.70
Male		08 (32%)	06 (43%)		14 (70%)	09 (47%)		09 (82%)	14 (50%)	
Female		17 (68%)	08 (57%)		06 (30%)	10 (53%)		02 (18%)	14 (50%)	
**Histological type**				0.517			0.139			0.891
Oligodendroglioma		09 (75%)	3 (25%)		09 (75%)	03 (25%)		04 (33%)	08 (67%)	
Astrocytoma		12 (63%)	07 (37%)		08 (42%)	11 (58%)		05 (26%)	14 (74%)	
Glioblastoma		04 (50%)	04 (50%)		03 (38%)	05 (62%)		02 (25%)	06 (75%)	
**Histological grade**				**0.001**			**<0.001**			0.760
**2**		02 (18%)	09 (82%)		0 (0%)	11 (100%)		04 (36%)	07 (64%)	
**3**		12 (92%)	01 (8%)		10 (77%)	03 (23%)		03 (23%)	10 (77%)	
**4**		11 (73%)	04 (27%)		10 (67%)	05 (33%)		04 (27%)	11 (73%)	
***IDH1-R132 mut***				0.153			0.325			0.376
Present		18 (72%)	07 (28%)		14 (56%)	11 (44%)		08 (32%)	17 (68%)	
Absent		07 (50%%)	07 (50%)		06 (43%)	08 (57%)		03 (21%)	11 (79%)	
***ATRX* mut/Loss**				0.261			0.17			0.380
Present		12 (57%)	09 (43%)		07 (33%)	14 (67%)		05 (24%)	16 (76%)	
Absent		13 (72%)	05 (28%)		13 (72%)	05 (28%)		06 (33%)	12 (67%)	
***Tp53* mut**				0.303			0.133			0.237
Present		09 (56%)	07 (44%)		06 (38%)	10 (62%0		06 (38%)	10 (62%)	
Absent		16 (70%)	07 (30%)		14 (61%)	09 (39%)		05 (22%)	18 (78%)	

**Table 5:** Correlation between autophagy-related genes in grade 2 gliomas.

	PI3K	BECLIN1	mTOR	AKT	PTEN	ULKI	ULK2	UVRAG	VPS34
**PI3K**	**-**	0.495	0.863	0.505	**0.824**	0.995	**0.731**	0.978	0.560
**BECLIN1**	0.495	**-**	**0.615**	0.110	**0.670**	0.505	0.462	0.495	0.275
**m-TOR**	0.863	**0.615**	**-**	0.357	**0.835**	**0.846**	**0.604**	0.896	0.473
**AKT**	0.505	0.110	0.357	-	0.434	0.500	0.247	0.489	0.055
**PTEN**	**0.824**	**0.670**	**0.835**	0.434	**-**	0.802	**0.714**	**0.846**	0.577
**ULK1**	0.995	0.505	**0.846**	0.500	**0.802**	**-**	**0.753**	0.956	0.516
**ULK2**	**0.731**	0.462	0.604	0.247	**0.714**	**0.753**	**-**	**0.670**	**0.626**
**UVRAG**	0.978	0.495	0.896	0.489	**0.846**	0.956	**0.670**	-	0.599
**VPS34**	0.560	0.275	0.473	0.055	0.577	0.516	**0.626**	0.599	-

The table displays the correlation coefficient (r) value for each pair of genes, with underlined values indicating statistically significant (*p* < 0.05) correlations between gene pairs.

**Table 6:** Correlation between autophagy-related genes in grade 3 gliomas.

	PI3K	BECLIN1	mTOR	AKT	PTEN	ULKI	ULK2	UVRAG	VPS34
**PI3K**	-	0.866	0.748	−0.094	0.988	0.674	0.781	0.747	0.192
**BECLIN1**	0.886	--	0.659	0.143	0.852	0.566	0.549	0.567	0.875
**mTOR**	0.748	0.659	-	−0.088	0.714	0.544	0.527	0.396	0.627
**AKT**	−0.094	0.143	−0.088	-	−0.104	−0.346	−0.538	−0.380	−0.204
**PTEN**	0.988	0.852	0.714	−0.104	-	0.621	0.753	0.735	0.889
**ULK1**	0.674	0.566	0.544	−0.346	0.621	-	0.742	0.784	0.652
**ULK2**	0.781	0.549	0.527	−0.538	0.753	0.742	-	0.814	0.715
**UVRAG**	0.747	0.567	0.396	−0.380	0.735	0.784	0.814	-	0.802
**VPS34**	0.912	0.875	0.627	−0.204	0.899	0.652	0.715	0.802	-

The table displays the correlation coefficient (r) value for each pair of genes, with underlined values indicating statistically significant (*p* < 0.05) correlations between gene pairs.

**Table 7:** Correlation between autophagy-related genes in grade 4 gliomas.

	PI3K	BECLIN1	mTOR	AKT	PTEN	ULKI	ULK2	UVRAG	VPS34
**PI3K**	-	0.721	0.461	0.479	0.646	0.943	0.782	0.893	0.600
**BECLIN1**	0.721	-	0.575	0.514	0.382	0.707	0.714	0.579	0.768
**mTOR**	0.461	0.575	-	0.504	0.557	0.496	0.386	0.432	0.411
**AKT**	0.479	0.514	0.504	-	0.443	0.571	0.486	0.361	0.464
**PTEN**	0.646	0.382	0.577	0.443	-	0.568	0.375	0.504	0.257
**ULK1**	0.943	0.707	0.496	0.571	0.568	-	0.811	0.886	0.643
**ULK2**	0.782	0.714	0.386	0.486	0.375	0.811	-	0.729	0.786
**UVRAG**	0.893	0.579	0.432	0.386	0.574	0.886	0.729	-	0.514
**VPS34**	0.600	0.768	0.411	0.464	0.257	0.643	0.786	0.514	-

The table displays the correlation coefficient (r) value for each pair of genes, with underlined values indicating statistically significant (*p* < 0.05) correlations between gene pairs.

**Table 8:** Names of target genes and their corresponding primer employed for mRNA quantification.

Genes	Forward Primer	Reverse Primer
Beclin-1	5′-AATGACTTTTTTCCTTAGGGGG-3′	5′-GTGGCTTTTGTGGATTTTTTCT-3′
m-TOR	5′-TGGGACAGCATGGAAGAATA-3′	5′-TGTTGTGCCAAGGAGAAGAG-3′
UVRAG	5′-CTGTTGCCCTTGGTTATACTGC-3′	5′-GATGATTTCTTCTGCTTGCTCC-3′
VPS34	5′-GCT GTC CTG GAA GAC CCA AT-3′	5′-TTC TCA CTG GCA AGG CCA AA-3′
PTEN	5′-CCAAGCTTATGACAGCCATCATC-3′	5′-CGCGGATCCTCAGACTTTTGTAA-3′
ULK1	5′-GGACACCATCAGGCTCTTCC-3′	5′-GAAGCCGAAGTCAGCGATCT-3′
ULK2	5′-TTCCTGCTCTAAGGGTTTGCTT-3′	5′-CCAGCGAGGGAGAACAACTG-3′
PI3K	5′-ATGCAAATTCAGTGCAAAGG-3′	5′-CGTGTAAACAGGTCAATGGC-3′
AKT	5′-GCAGCACGTGTACGAGAAGA-3′	5′ -GGTGTCAGTCTCCGACGTG-3′

## Data Availability

Data will be provided upon request.

## References

[1] Ferlay J., Soerjomataram I., Ervik M., Dikshit R., Eser S., Mathers C., Rebelo M., Parkin D.M., Forman D., Bray F. (2013). GLOBOCAN 2012 v1.0, Cancer Incidence and Mortality Worldwide: IARC CancerBase No. 11 [Internet].

[2] Ostrom Q.T., Gittleman H., Xu J., Kromer C., Wolinsky Y., Kruchko C., Barnholtz-Sloan J.S. (2016). CBTRUS statistical report: Primary brain and other central nervous system tumors diagnosed in the United States in 2009–2013. Neuro-Oncol..

[3] Ostrom Q.T., Price M., Neff C., Cioffi G., Waite K.A., Kruchko C., Barnholtz-Sloan J.S. (2022). CBTRUS statistical report: Primary brain and other central nervous system tumors diagnosed in the United States in 2015–2019. Neuro-Oncol..

[4] Louis D.N., Perry A., Wesseling P., Brat D.J., Cree I.A., Figarella-Branger D., Hawkins C., Ng H., Pfister S.M., Reifenberger G. (2021). The 2021 WHO classification of tumors of the central nervous system: A summary. Neuro-Oncol..

[5] Fisher J., Schwartzbaum J., Wrensch M., Wiemels J.L. (2007). Epidemiology of brain tumors. Neurol. Clin..

[6] Krakstad C., Chekenya M. (2010). Survival signalling and apoptosis resistance in glioblastomas: Opportunities for targeted therapeutics. Mol. Cancer.

[7] Miyashita T., Krajewski S., Krajewska M., Wang H.G., Lin H., Liebermann D.A., Hoffman B., Reed J.C. (1994). Tumor suppressor p53 is a regulator of bcl-2 and bax gene expression in vitro and in vivo. Oncogene.

[8] Liu F., Liu D., Yang Y., Zhao S. (2013). Effect of autophagy inhibition on chemotherapy-induced apoptosis in A549 lung cancer cells. Oncol. Lett..

[9] Condello M., Mancini G., Meschini S. (2020). The exploitation of liposomes in the inhibition of autophagy to defeat drug resistance. Front. Pharmacol..

[10] Levine B., Klionsky D.J. (2004). Development by self-digestion: Molecular mechanisms and biological functions of autophagy. Dev. Cell.

[11] Murrow L., Debnath J. (2013). Autophagy as a stress-response and quality-control mechanism: Implications for cell injury and human disease. Annu. Rev. Pathol. Mech. Dis..

[12] Li Z., Bao S., Wu Q., Wang H., Eyler C., Sathornsumetee S., Shi Q., Cao Y., Lathia J., McLendon R.E. (2009). Hypoxia-inducible factors regulate tumorigenic capacity of glioma stem cells. Cancer Cell.

[13] Sun Y., Xing X., Liu Q., Wang Z., Xin Y., Zhang P., Hu C., Liu Y. (2015). Hypoxia-induced autophagy reduces radiosensitivity by the HIF-1α/miR-210/Bcl-2 pathway in colon cancer cells. Int. J. Oncol..

[14] Wu H.-M., Jiang Z.-F., Ding P.-S., Shao L.-J., Liu R.-Y. (2015). Hypoxia-induced autophagy mediates cisplatin resistance in lung cancer cells. Sci. Rep..

[15] Denton D., Nicolson S., Kumar S. (2012). Cell death by autophagy: Facts and apparent artefacts. Cell Death Differ..

[16] Lu N., Li X., Tan R., An J., Cai Z., Hu X., Wang F., Wang H., Lu C., Lu H. (2018). HIF-1α/Beclin1-mediated autophagy is involved in neuroprotection induced by hypoxic preconditioning. J. Mol. Neurosci..

[17] Menon M.B., Dhamija S. (2018). Beclin 1 phosphorylation–at the center of autophagy regulation. Front. Cell Dev. Biol..

[18] Pirtoli L., Cevenini G., Tini P., Vannini M., Oliveri G., Marsili S., Mourmouras V., Rubino G., Miracco C. (2009). The prognostic role of Beclin 1 protein expression in high-grade gliomas. Autophagy.

[19] Apel A., Herr I., Schwarz H., Rodemann H.P., Mayer A. (2008). Blocked autophagy sensitizes resistant carcinoma cells to radiation therapy. Cancer Res..

[20] Wu J., Lei Z., Yu J. (2015). Hypoxia induces autophagy in human vascular endothelial cells in a hypoxia-inducible factor 1-dependent manner. Mol. Med. Rep..

[21] Wei J., Zhu K., Yang Z., Zhou Y., Xia Z., Ren J., Zhao Y., Wu G., Liu C. (2023). Hypoxia-induced autophagy is involved in radioresistance via HIF1A-associated beclin-1 in glioblastoma multiforme. Heliyon.

[22] Park S.Y., Sun E.G., Lee Y., Kim M.S., Kim J.H., Kim W.J., Jung J.Y. (2018). Autophagy induction plays a protective role against hypoxic stress in human dental pulp cells. J. Cell. Biochem..

[23] White E. (2012). Deconvoluting the context-dependent role for autophagy in cancer. Nat. Rev. Cancer.

[24] Mathew R., Karp C.M., Beaudoin B., Vuong N., Chen G., Chen H.-Y., Bray K., Reddy A., Bhanot G., Gelinas C. (2009). Autophagy suppresses tumorigenesis through elimination of p62. Cell.

[25] Karantza-Wadsworth V., Patel S., Kravchuk O., Chen G., Mathew R., Jin S., White E. (2007). Autophagy mitigates metabolic stress and genome damage in mammary tumorigenesis. Genes Dev..

[26] Mathew R., Kongara S., Beaudoin B., Karp C.M., Bray K., Degenhardt K., Chen G., Jin S., White E. (2007). Autophagy suppresses tumor progression by limiting chromosomal instability. Genes Dev..

[27] Azad M.B., Chen Y., Henson E.S., Cizeau J., McMillan-Ward E., Israels S.J., Gibson S.B. (2008). Hypoxia induces autophagic cell death in apoptosis-competent cells through a mechanism involving BNIP3. Autophagy.

[28] Bellot G., Garcia-Medina R., Gounon P., Chiche J., Roux D., Pouysségur J., Mazure N.M. (2009). Hypoxia-induced autophagy is mediated through hypoxia-inducible factor induction of BNIP3 and BNIP3L via their BH3 domains. Mol. Cell. Biol..

[29] Mohammed S.M., Elesawy Y.F., Abd El Aziz A.M., Khairy R.A. (2022). The Pathological Evaluation of Autophagy-Related Protein (LC3B) and Its Association with the Infiltration of Immune Cells in Glioma. Asian Pac. J. Cancer Prev. APJCP.

[30] Tamrakar S., Yashiro M., Kawashima T., Uda T., Terakawa Y., Kuwae Y., Ohsawa M., Ohata K. (2019). Clinicopathological significance of autophagy-related proteins and its association with genetic alterations in gliomas. Anticancer. Res..

[31] Mathew R., Karantza-Wadsworth V., White E. (2007). Role of autophagy in cancer. Nat. Rev. Cancer.

[32] Das C.K., Mandal M., Kögel D. (2018). Pro-survival autophagy and cancer cell resistance to therapy. Cancer Metastasis Rev..

[33] Ivankovic D., Chau K.Y., Schapira A.H., Gegg M.E. (2016). Mitochondrial and lysosomal biogenesis are activated following PINK 1/parkin-mediated mitophagy. J. Neurochem..

[34] Deng D., Luo K., Liu H., Nie X., Xue L., Wang R., Xu Y., Cui J., Shao N., Zhi F. (2019). p62 acts as an oncogene and is targeted by miR-124-3p in glioma. Cancer Cell Int..

[35] Rzymski T., Milani M., Pike L., Buffa F., Mellor H., Winchester L., Pires I., Hammond E., Ragoussis I., Harris A. (2010). Regulation of autophagy by ATF4 in response to severe hypoxia. Oncogene.

[36] Crighton D., Wilkinson S., O’Prey J., Syed N., Smith P., Harrison P.R., Gasco M., Garrone O., Crook T., Ryan K.M. (2006). DRAM, a p53-induced modulator of autophagy, is critical for apoptosis. Cell.

[37] Geng J., Zhang R., Yuan X., Xu H., Zhu Z., Wang X., Wang Y., Xu G., Guo W., Wu J. (2020). DRAM1 plays a tumor suppressor role in NSCLC cells by promoting lysosomal degradation of EGFR. Cell Death Dis..

[38] Galavotti S., Bartesaghi S., Faccenda D., Shaked-Rabi M., Sanzone S., McEvoy A., Dinsdale D., Condorelli F., Brandner S., Campanella M. (2013). The autophagy-associated factors DRAM1 and p62 regulate cell migration and invasion in glioblastoma stem cells. Oncogene.

[39] Ronan B., Flamand O., Vescovi L., Dureuil C., Durand L., Fassy F., Bachelot M.-F., Lamberton A., Mathieu M., Bertrand T. (2014). A highly potent and selective Vps34 inhibitor alters vesicle trafficking and autophagy. Nat. Chem. Biol..

[40] Dyczynski M., Yu Y., Otrocka M., Parpal S., Braga T., Henley A.B., Zazzi H., Lerner M., Wennerberg K., Viklund J. (2018). Targeting autophagy by small molecule inhibitors of vacuolar protein sorting 34 (Vps34) improves the sensitivity of breast cancer cells to Sunitinib. Cancer Lett..

[41] Marsh T., Debnath J. (2015). Ironing out VPS34 inhibition. Nat. Cell Biol..

[42] Hennessy B.T., Smith D.L., Ram P.T., Lu Y., Mills G.B. (2005). Exploiting the PI3K/AKT pathway for cancer drug discovery. Nat. Rev. Drug Discov..

[43] Hu M., Zhu S., Xiong S., Xue X., Zhou X. (2019). MicroRNAs and the PTEN/PI3K/Akt pathway in gastric cancer. Oncol. Rep..

[44] Jiang N., Dai Q., Su X., Fu J., Feng X., Peng J. (2020). Role of PI3K/AKT pathway in cancer: The framework of malignant behavior. Mol. Biol. Rep..

[45] Brennan C.W., Verhaak R.G., McKenna A., Campos B., Noushmehr H., Salama S.R., Zheng S., Chakravarty D., Sanborn J.Z., Berman S.H. (2013). The somatic genomic landscape of glioblastoma. Cell.

[46] Errafiy R., Aguado C., Ghislat G., Esteve J.M., Gil A., Loutfi M., Knecht E. (2013). PTEN increases autophagy and inhibits the ubiquitin-proteasome pathway in glioma cells independently of its lipid phosphatase activity. PLoS ONE.

[47] Giotta Lucifero A., Luzzi S. (2022). Immune landscape in PTEN-related glioma microenvironment: A bioinformatic analysis. Brain Sci..

[48] Simpson L., Parsons R. (2001). PTEN: Life as a tumor suppressor. Exp. Cell Res..

[49] Morris D.H., Yip C.K., Shi Y., Chait B.T., Wang Q.J. (2015). Beclin 1-Vps34 complex architecture: Understanding the nuts and bolts of therapeutic targets. Front. Biol..

[50] Schläfli A., Berezowska S., Adams O., Langer R., Tschan M. (2015). Reliable LC3 and p62 autophagy marker detection in formalin fixed paraffin embedded human tissue by immunohistochemistry. Eur. J. Histochem. EJH.

[51] Ladoire S., Chaba K., Martins I., Sukkurwala A.Q., Adjemian S., Michaud M., Poirier-Colame V., Andreiuolo F., Galluzzi L., White E. (2012). Immunohistochemical detection of cytoplasmic LC3 puncta in human cancer specimens. Autophagy.

[52] Jiang T., Wu Z. (2018). Immunohistochemical assessment of autophagic protein LC3B and p62 levels in glioma patients. Int. J. Clin. Exp. Pathol..

[53] Wudu M., Ren H., Hui L., Jiang J., Zhang S., Xu Y., Wang Q., Su H., Jiang X., Dao R. (2019). DRAM2 acts as an oncogene in non-small cell lung cancer and suppresses the expression of p53. J. Exp. Clin. Cancer Res..

[54] Masuda G., Yashiro M., Kitayama K., Miki Y., Kasashima H., Kinoshita H., Morisaki T., Fukuoka T., Hasegawa T., Sakurai K. (2016). Clinicopathological correlations of autophagy-related proteins LC3, Beclin 1 and p62 in gastric cancer. Anticancer. Res..

[55] Ahmed K., Sheikh A., Fatima S., Haider G., Ghias K., Abbas F., Mughal N., Abidi S.H. (2022). Detection and characterization of latency stage of EBV and histopathological analysis of prostatic adenocarcinoma tissues. Sci. Rep..

